# Evaluation of the Clinical Efficacy of the Treatment of Overweight and Obesity in Type 2 Diabetes Mellitus by the Telemedicine Management System Based on the Internet of Things Technology

**DOI:** 10.1155/2022/8149515

**Published:** 2022-06-22

**Authors:** Kaisang Lin, Wei Zhang, Fei He, Jie Shen

**Affiliations:** ^1^Department of Endocrinology, Municipal Hospital Affiliated to Taizhou University, Taizhou 318000, China; ^2^Department of Endocrinology, The Third Affiliated Hospital of Southern Medical University, Guangzhou 510000, China

## Abstract

**Objective:**

To explore the application value of medical intelligent electronic system under the background of Internet of Things in the clinical study of the treatment of overweight/obesity in type 2 diabetes mellitus (T2DM) with empagliflozin combined with liraglutide; 50 overweight and obese adult T2DM patients in our hospital were randomly divided into the combined group and the control group, 25 cases in each group. The control group was treated with liraglutide alone, while the combined group was treated with empagliflozin on the basis of liraglutide. Based on the Internet of Things technology, with diabetes management as the core, the functions of information collection, transmission, and storage of T2DM patients are realized. Doctors pass the diabetes management plan to T2DM patients through the platform, supervise the implementation, and finally compare the clinical efficacy of the two groups.

**Results:**

Compared with before treatment, the body mass index (BMI), fasting blood glucose (FPG), postprandial blood glucose (2hPG), glycosylated hemoglobin (HbAlc), islet beta cell secretion function index (HOMA-*β*), islet resistance index (HOMA-IR), total cholesterol (TC), and triglyceride (TG) in both groups decreased significantly after treatment. After combined treatment, systolic blood pressure (SBP), diastolic blood pressure (DBP), FPG, 2hPG, HbA1c, and HOMA-IR in the combined group were significantly lower than those in the control group (*P* < 0.05). Hypoglycemia occurred in both groups, with 2 cases in the control group and 4 cases in the combined group.

**Conclusion:**

The telemedicine management system based on Internet of Things technology can improve patients' self-management ability and provide a new choice for individualized treatment of overweight/obesity T2DM patients. The combination therapy of empagliflozin and liraglutide can effectively reduce blood sugar, weight, blood pressure, blood lipid, and hypoglycemia and effectively improve insulin resistance and secretion function of islet *β* cells in T2DM patients.

## 1. Introduction

Type 2 diabetes (T2DM) is a complex metabolic disease. Apart from pathoglycemia, T2DM occurs with the abnormalities of some metabolic indexes, including weight, blood pressure, and blood fat. Epidemiological investigation demonstrates that 113.9 million people among Chinese adults suffer from diabetes and its complications [1]. In recent years, the minimization of the risk of weight gain and the priority selection of blood sugar-reducing plans with multiple benefits including weight loss are emphasized among the recommended selections of the treatment plans for diabetes patients in all domestic and foreign authoritative guides [2]. Liraglutide is a new synthetical glucagon-like peptide-1 analog (GLP-1RA), which can effectively reduce blood sugar level. Besides, it is featured with low risk in hypoglycemia, weight loss, and low incidence of adverse events [3]. Empagliflozin refers to a sodium-glucose cotransporter 2 inhibitor (SGLT2i). According to current studies, SGLT2i can not only reduce blood sugar level effectively but also result in weight loss significantly. What's more, it plays a role in reducing blood pressure and proteinuria [4]. However, there are still few reports related to the joint treatment of liraglutide and empagliflozin.

Internet of things (IoT) refers to the exchange of information among things by adopting sensors to connect things according to certain communication protocols [5–7]. As an interconnected network of communication, IoT utilizes radio frequency identification (RFID), infrared sensors, global positioning system (GPS), and other sensors to identify, locate, track, monitor, and manage objects. IoT adopts advanced sensor technologies to collect various natural information, including force, heat, light, electricity, and sound. Besides, it realizes the intelligent identification and management of information by monitoring process, object identification, or positioning. With the increase in Chinese online users and the perfection of network facilities, network-based remote medical treatment management can open up a broad market for the development of the medical treatment industry in the future and improve the overall medical treatment level. Diabetes remote medical treatment management system is an essential branch of remote medical treatment. Currently, a health management system specially for diabetes patients emerges domestically and abroad. Combined with information technology (IT) and healthcare, the health management system adopts electronic network technology to access to medical treatment service system of health management service anytime and anywhere. Diabetes remote medical treatment management system utilizes bioinformation detectors to monitor patients' body signals and health information. In addition, it utilizes communication to transmit data centers to hospitals and return them to patients after completing data analysis [8, 9]. Based on the medical information and data offered by data centers and the guidance of medical treatment groups, patients conduct self-constraint management. In addition, the management system can evaluate therapeutic effects, and it is suitable for subhealth population and healthy population in need of health management. The above populations are offered the corresponding healthcare services by the management system.

In this study, 50 patients with T2DM who received diabetes treatment in our hospital from January 2019 to July 2020 were selected as research objects, and they were randomly divided into control group and combined group. We constructed a diabetes telemedicine management system based on Internet of Things technology, explored its clinical application effect in the treatment of overweight and obesity T2DM with Englising and liraglutide, and discussed the effectiveness and practicability of the system.

## 2. Materials and Methods

### 2.1. General Data

The information about the research objects was as follows: a total of 50 T2DM patients receiving diabetes treatment in hospital between January 2019 and July 2020 was selected as the research objects. All selected cases were randomly divided into control group and joint group, each of which included 25 cases.  Table 1 shows the general data on all cases below. Patients in two groups were all monitored by IoT technology-based diabetes management information system. Besides, the implementation of the research had been approved by Hospital Medical Ethics Committee. Patients as well as their family members had been informed of the research and signed the informed consent forms.

The patients were included in the research based on the following standards: (A) all patients' symptoms met the diagnostic standards for T2DM stipulated by World Health Organization [10]. (B) patients' body mass index (BMI) levels were equal to or higher than 24 kg/m^2^, and (C) patients' glycosylated hemoglobin (HbA1c) levels ranged between 7.0% and 10.0%.

The patients were excluded from the research based on the following standards: (A) patients did not suffer from T2DM; (B) patients suffered from serious heart, liver, and kidney dysfunctions; (C) patients suffered from the abnormalities of complicated immune system and blood coagulation functions; (D) patients suffered from allergic constitution; and (E) patients had psychiatric history or psychiatric disorders.

### 2.2. Research Methods

The methods adopted for patients in the control group were as follows: the initial dose of liraglutide (0.6 mg) was injected with liraglutide needles (Denmark Novo Nordisk Company). Subcutaneous injection was performed on patients once every day. After 1 week, the injection dose was increased to 1.2 mg, and the injection was performed once every day.

The methods adopted for patients in the joint group were as follows: based on the adoption of liraglutide needles, 10 mg of empagliflozin (Eli Lilly and Company) was taken orally once every day.

The treatment course for the two groups lasted for 3 months. Follow-up visits occurred once every 4 weeks. During each visit, blood sugar level was monitored, and hypoglycemia as well as other adverse reactions was recorded. In the treatment process, the blood sugar level being equal to or lower than 3.9 mmoL/L was defined as hypoglycemia event standard.

### 2.3. Observation Indexes

IoT technology-based diabetes management information system was utilized for follow-up visits. Based on the observation of weight and height values before and after treatment, BMI values were calculated. Systolic blood pressure (SBP) and diastolic blood pressure (DBP) were measured. Besides, early morning fasting venous blood in the early morning and 2 hours after breakfast were extracted to detect fasting plasma glucose (FPG) (mmoL/L), fasting insulin (FINS) (*μ*U/mL), 2 h postprandial blood glucose (2hPG), glycosylated hemoglobin (HbAlc) (%), total cholesterol (TC), triglyceride (TG), and low-density lipoprotein (LDL). In addition, insulin resistance (HOMA-IR) and islet cell *β* secretion function index (HOMA-*β*) were calculated. The calculation equations are as follows:

(1) $$\begin{array}{c} HOMA-IR=\frac{FPG\;\times FINS}{22.5},\\ HOMA-\beta =\frac{20\times FINS}{FPG}-3.5. \end{array}$$

Adverse reaction indexes were observed, including the incidence of hypoglycemia, gastrointestinal reaction, rash, and urine leukocyte. Besides, the reasons for the loss of follow-up visits were recorded.

### 2.4. Framework of IoT Technology-Based Diabetes Remote Medical Treatment Management System

IoT technology-based diabetes remote medical treatment management system was a specific application of intelligent medical treatment system. The main functions of the system included the assistance in diabetes management by collecting and processing patients' information, the lowering of the barriers for diabetes patients' self-management, and the enhancement of the effectiveness in managing patients by doctors [11].

**Figure 1:**
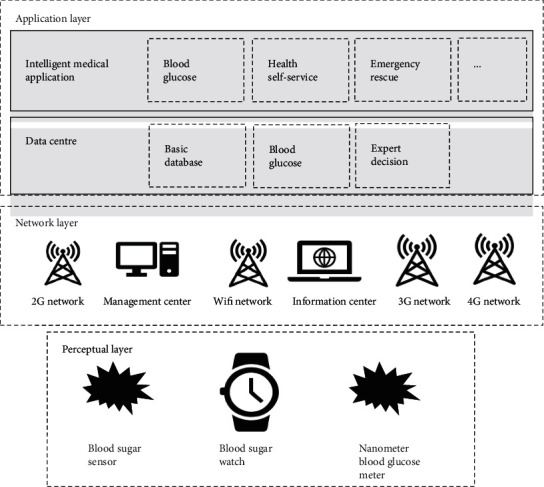
Framework of the IoT technology-based diabetes remote medical treatment management system.

IoT technology-based diabetes remote medical treatment management system consisted mainly of three components. The first one was the perception layer, whose main functions included the collection of intelligent glucometer and other signs and the realization of the digital and automatic acquisition of management data. The second one was the network layer, whose main function was the transmission of the data acquired by the perception layer and feedback information of the application layer. The third one was the application layer, which was responsible mainly for processing the data network layer transmitted and formulating management and guidance plans by analysis and processing. The system could achieve the comprehensive evaluation of patients by acquiring, transmitting, and processing diabetes management information of patients.  Figure 1 shows the framework of the IoT technology-based diabetes remote medical treatment management system.

### 2.5. Function Needs of the Diabetes Remote Medical Treatment Management System

The main functions of diabetes remote medical treatment management system were the analysis and processing of the data collected and input into the system by the information collection system and the subsequent integration, extraction, and feedback of information according to diabetes management procedures. Firstly, the system needed to evaluate patients' current physical health to acquire comprehensive evaluation and feedback. Secondly, the system provided doctors with the overall report of monitoring data, identified hazard information factors, and assisted doctors in fast diagnosis and processing. Thirdly, the system needed to possess the function of instant communication to enable patients to contact with doctors anytime and anywhere and to send data and information. Therefore, diabetes remote medical treatment management system should include at least the following 6 functions.

The first one was archives management of personal health information about diabetes patients. The second one was comprehensive evaluation of patients' health status. The third one was reminder management. The fourth one was remote interrogation. The fifth one was emergency warning. The sixth one was diagnosis and treatment plan sending.

### 2.6. Design of the Diabetes Remote Medical Treatment Management System

**Figure 2:**
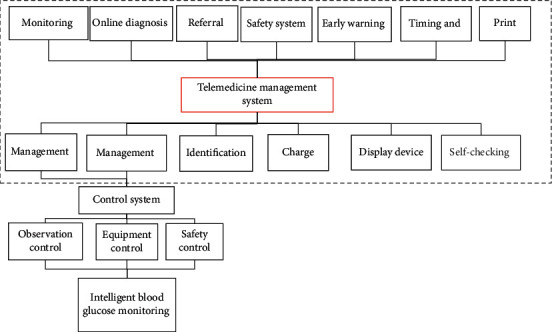
Composition of the diabetes remote medical treatment management system.

Diabetes remote medical treatment management system designed in the research consisted mainly of two components. One of them was the real-time collection management of patients' information. The component system remotely collected the blood sugar values at critical periods, including those in fasting, before and after three meals, and at night, and then transmitted the collected data to the management system by electronic network means. Another component system was the analysis and evaluation of blood sugar and other management data. This component system compared the acquired data with the management targets of patients and then assessed whether daily management reached the standard. Abnormal data were reported to doctors in time. The analysis of the data on patients before and after abnormalities provided doctors with detailed diagnosis basis. Besides, the system could also identify the blood sugar in abnormal ranges and actively inform patients about the solutions.  Figure 2 shows the composition of diabetes remote medical treatment management system.

### 2.7. Process of the IoT Technology-Based Diabetes Remote Medical Treatment Management System

**Figure 3:**
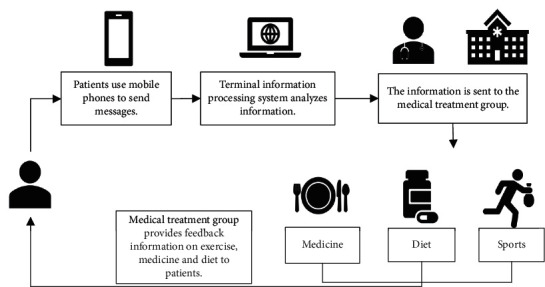
Process of the IoT technology-based diabetes remote medical treatment management system.

In the research, the free obtained MyGlucoHealtH glucometer was adopted to upload blood sugar values automatically. Blood sugar values were measured at least twice each day and twice to three times every week. After being connected with computers by data lines, glucometer could upload blood sugar data to the central database automatically. Meanwhile, patients could input relevant health profiles, including diet, exercise, blood pressure, and weight on diabetes remote medical treatment management system website. Medical treatment group logged in to the website to analyze blood sugar levels and caloric intake as well as consumption once every two weeks. After that, the group members offered patients their suggestions by leaving web page messages or phone calls. In addition, the group supervised patients' self-monitoring of blood sugar and the implementation of diet and exercise plans. If patients did not upload blood sugar data within 1 week, they would receive web page messages or short message reminders.  Figure 3 displays the process of IoT technology-based diabetes remote medical treatment management system.

### 2.8. Mobile Phone Diabetes Remote Medical Treatment Management System

**Figure 4:**
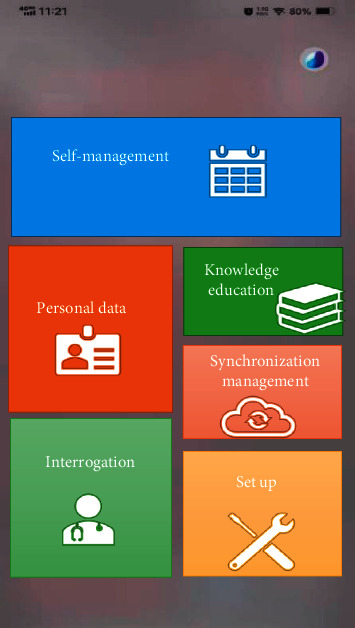
Main framework interface of mobile phone diabetes remote medical treatment management software.

In the experiment, Java language development was adopted to achieve the operation of mobile phone diabetes remote medical treatment management system. The development environment was the Android 4.2 mobile operating system. In the process of design, the interaction between users and system needed to be realized from the perspective of users. The main framework was divided mainly into four modules, including self-management, personal data, knowledge education, and interrogation.  Figure 4 presents all the four modules of the framework.

**Figure 5:**
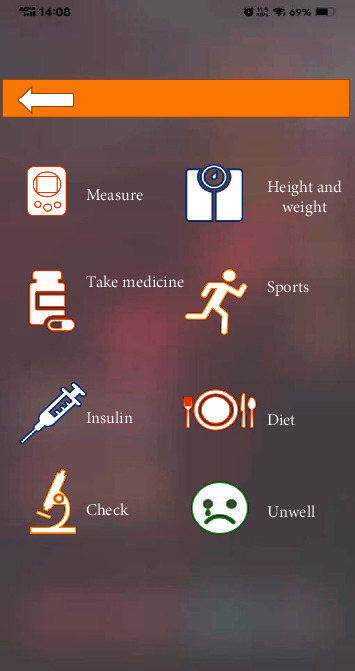
Self-management interface.

**Figure 6:**
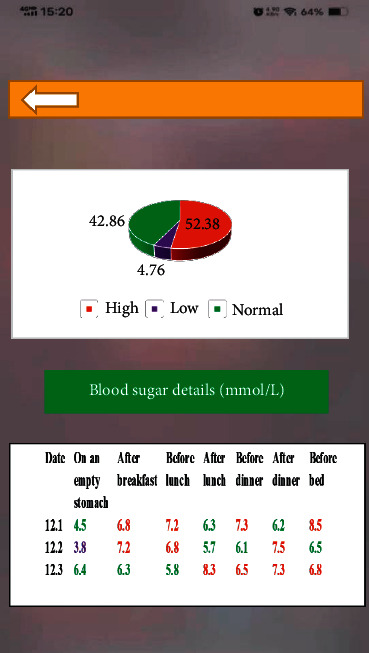
Self-management interface—blood sugar analysis.

Self-management module was adopted mainly to record the process of diabetes patient management. The module included records and reminder. Blood sugar monitoring and medication reminders could be set up in advance. Besides, exercise, diet, and medication management could be recorded in this module. If patients completed the recording, the system would automatically assess whether the current blood sugar values of users were normal according to the targets set by patients. After that, the information would be automatically synchronized to users' personal profiles. If abnormalities occurred, the system would check the causes automatically for doctors. Figures 5 and 6 demonstrate self-management interface.

**Figure 7:**
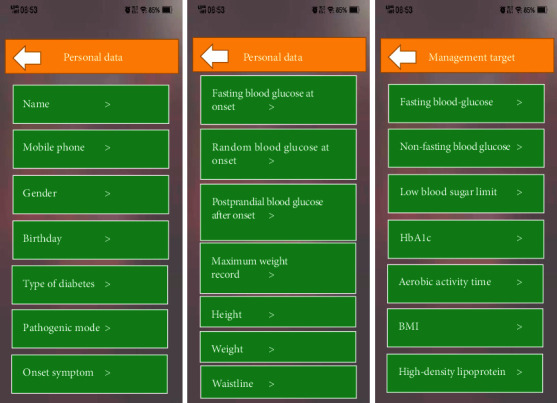
Personal data interface. *Note.* BMI refers to body mass index, and HbA1c denotes glycosylated hemoglobin.

Personal data module mainly included patients' personal data, information, and management target. During the follow-up visits, doctors could query patients' medical history and management targets in real time. In case of emergency, doctors could locate patients' geographical location according to GPS on users' mobile phones and then perform rescue calls.  Figure 7 shows personal data interface.

**Figure 8:**
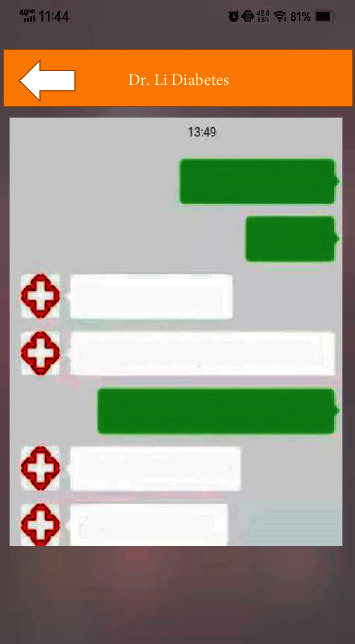
Interrogation interface.

Interrogation system offered communication function for doctors and patients at any time. Real-time communication could be achieved through text, picture, voice, and video, which enabled patients to consult with doctors for help anytime and anywhere.  Figure 8 shows interrogation interface.

### 2.9. Doctor Remote Medical Treatment Management Platform

Doctor remote medical treatment management platform focused mainly on diabetes patient's mobile phone management, and it consisted of three components, including patient grouping, patient information management, and management log.

**Figure 9:**
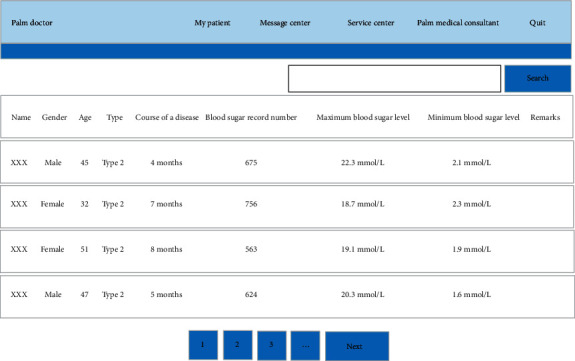
Patient grouping interface.

To enhance doctors' management efficiency, patients were divided into different groups. According to the results of system test, each doctor could manage about 50 diabetes patients each day. Patient grouping could promote the rapport between doctors and patients and improve the relationship between doctors and patients.  Figure 9 demonstrates the patient grouping interface.

**Figure 10:**
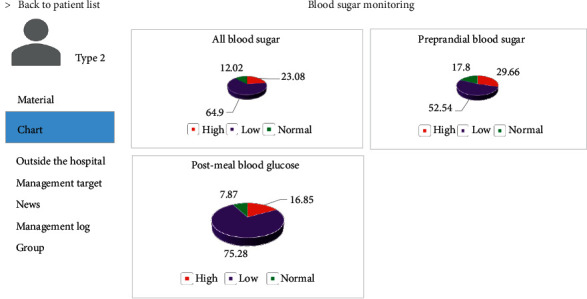
Patient information management—blood sugar monitoring interface.

**Figure 11:**
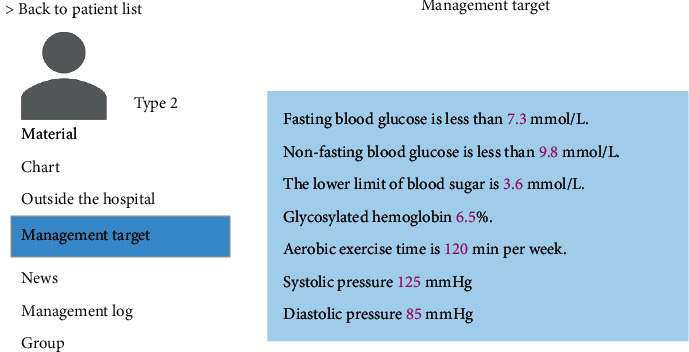
Patient information management—target management interface.

Patient information management included overall patient management, integration, and analysis of the data offered by patients and blood sugar monitoring.  Figure 10 displays patient information management—blood sugar monitoring interface. When blood sugar abnormality of patients occurred, the relevant information about outside hospital management of patients, including diet, medication, and target management, could be checked timely, and reasonable treatment plans were formulated.  Figure 11 demonstrates the patient information management—target management interface.

**Figure 12:**
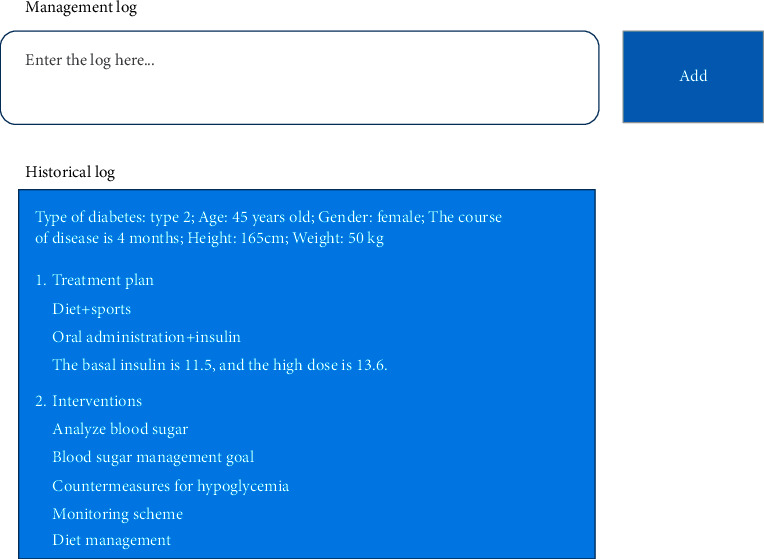
Patient information management—management log interface.

Based on the follow-up visits, doctors recorded the basic information of patients in management log, whose main functions included the adjustment of patient management plans and management targets.  Figure 12 displays the patient information management—management log interface.

### 2.10. Statistical Methods

Statistical product and service solutions (SPSS) 25.0 was adopted for data entry and statistical analysis. All measurement data were expressed by mean ± deviation ($\bar{x}\pm s$). The comparison between the two groups was tested by the independent sample *t*, and the comparison of the same group before and after treatment was tested by paired sample *t*. *P* < 0.05 indicates that the differences showed statistical significance.

## 3. Results

### 3.1. Comparison of General Data and Biochemical Indexes in Two Groups before and after Treatment

After the treatment, weight, BMI, FPG, 2hPG, HbA1c, HOMA-*β*, HOMA-IR, TC, and TG in two groups were all obviously reduced compared with those in each group before treatment, and the differences showed statistical significance (*P* < 0.05). In contrast, LDL after the treatment was not significantly different from that before treatment in each group. Besides, SBP and DBP in observation group after the treatment were decreased compared with those in the group before the treatment (*P* < 0.05). In control group, the comparison of SBP and DBP after and before the treatment indicated that the differences showed no statistical significance. Compared with those in control group before the treatment, weight, BMI, SBP, DBP, FPG, 2hPG, HbA1c, and HOMA-IR in observation group were further reduced after the treatment, and the differences showed statistical significance (*P* < 0.05). In contrast, the comparison between HOMA-*β*, TC, and TG in control group before the treatment and those in observation group after the treatment demonstrated no significant differences.  Table 2 displays the comparison of each index in two groups before and after the treatment.

### 3.2. Comparison of Adverse Reactions between Patients in Two Groups

Hypoglycemia events occurred in both groups. In the control group, 2 patients suffered from hypoglycemia. Besides, 4 cases in the joint group reported hypoglycemia. The comparison of the incidence of hypoglycemia in two groups revealed that the differences showed no statistical significance (*P* > 0.05). The main adverse reaction in the two groups was gastrointestinal reaction, which was usually improved by themselves or symptomatic treatment within 1 to 2 weeks. Besides, urinary tract infection symptoms, including frequent micturition, urgent micturition, dysuria, and urinary tract fever, were not detected in the joint treatment group.

### 3.3. Reasons for the Loss of Follow-Up Visits of Patients in Two Groups

**Figure 13:**
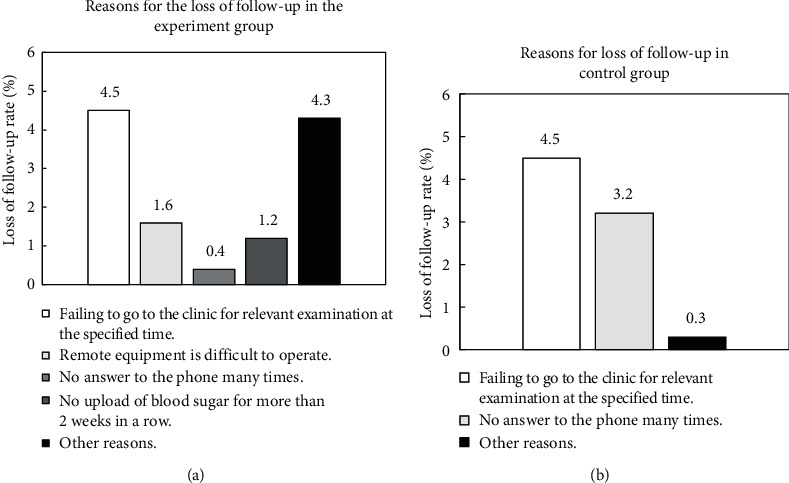
Reasons for loss of follow-up visits of patients in two groups. (a) The reasons for loss of follow-up visits in the experimental group and (b) the reasons for loss of follow-up visits in the control group.

At the end of the experiment, 3 patients in the experimental group lost follow-up visits, and 2 patients in the control group lost follow-up visits. The follow-up loss rate in the two groups reached 12% and 8%, respectively.  Figure 13 illustrates the reasons for the loss of follow-up visits. By the end of the experiment, about 75% of patients in the experimental group insisted on blood sugar monitoring for 2 to 3 days each week.

## 4. Discussion

According to the domestic growing incidence of diabetes day by day and the high incidence of diabetes complications, the management of diabetes is always a difficult problem in the medical field. Diagnosis is emphasized in traditional diabetes management, while little attention is paid to management. As a result, management effects are so poor that the growing needs of diabetes patients cannot be met. Based on the analysis, the fundamental problem in diabetes management issue lies in patients' self-management. The key to addressing patients' self-management problems lies in the promotion of relevant knowledge, including how to monitor blood sugar, plan diet, and engage in exercise [12–15]. IoT technology-based diabetes remote medical treatment management system offered opportunities to solve the above problems. Intelligent glucometer in the perception layer could continuously monitor patients' blood sugar and acquire key data adopted to evaluate patients' management levels. Diabetes remote medical treatment management system could utilize IT and communication technology to collate and analyze patients' management data and automatically generate the next management plan. Patients only needed to execute the plan directly according to feedback [16–19]. From the perspectives of both doctors and patients, diabetes remote medical treatment management system adopted IoT technology to tackle the problem of monitoring patients' physiological indexes. Besides, it enhanced doctors' management efficiency to enable the high-efficient interconnection and interaction between doctors and patients and to further solve the current difficulty in diabetes management [20–22]. In the research, remote medical treatment management system with mobile phones as terminal carriers was studied and designed according to patients' needs. In addition, its management functions were modularized. Remote medical treatment management platform was designed and operated from the perspective of doctors' needs to achieve real-time and convenient communication between doctors and patients.

Obesity/overweight is one of the major hazard factors of diabetes. Numerous free fatty acids secreted by adipose tissue damage the sensitivity and secretion function of islet *β* cells. The damaged sensitivity and secretion function not only result in and aggravate insulin resistance but also enhance the risks of cardiovascular death and all-cause mortality. According to relevant studies, the weight loss of 10 kg can reduce cardiovascular mortality among diabetes patients by about 25% [23]. Insulin resistance and progressive decline of islet cell *β* functions are the main pathological and physiological mechanisms of diabetes. GLP-1RA possesses the function of two-way regulation of islet *α*/*β* cells. In the human body, GLP-1RA promotes the secretion of insulin and inhibits glucagon in the form of glucose concentration dependence. Besides, it delays gastrointestinal emptying, inhibits appestat, and reduces the intake of food and heat. As a result, patients' weight and blood sugar are reduced [24, 25]. The results of the series studies conducted by Dardano et al. [26] demonstrated that liraglutide could reduce diabetes patients' HbA1c levels by 0.8–1.5%, which was consistent with the result of the research. In addition, in vitro studies on animals and humans verified [27, 28] that GLP-1RA could increase the number of *β* cells by promoting the rebirth and differentiation of *β* cells and reducing their apoptosis. It could protect islet *β* cells. In the research, islet cell *β* secretion indexes HOMA-*β* in the control group were enhanced after the treatment by liraglutide, while HOMA-IR levels were reduced compared with those before the treatment. The results indicated that liraglutide could effectively promote the recovery of islet *β* function and improve insulin resistance indexes of overweight/obesity patients.

After the treatment by empagliflozin, weight, BMI, FBG as well as 2hPG, HbA1c, and HOMA-IR levels of patients in the joint group were further decreased compared with those in the control group, which might be caused by the synergetic effect generated by the mutual complementation between GLP-1RA and SGLT2i through different action mechanisms. In physiological conditions, SGLT2 cotransporter proteins on proximal renal tubules got involved in the resorption of 90% glucose in renal glomerulus filtered solution. In addition, SGLT2 inhibitor could inhibit the resorption of filtered glucose by renal tubules specifically to facilitate glycosuria discharge. In addition, it could generate mild osmotic diuresis and show the function similar to the extra consumption of calories by the body. In this case, weight and total fat were reduced, and its blood sugar-reducing effects became independent from the islet cell *β* function of patients themselves and the impacts of insulin resistance [29]. Based on the study, Li et al. [30] found out that empagliflozin could increase the browning of white adipose tissue and further enhance fat consumption. What's more, it could alleviate inflammatory reactions and insulin resistance caused by obesity by activating M2 macrophages. Consequently, it showed significant effects in weight loss and blood sugar reduction.

In addition, SBP and DBP of patients in the observation group were both obviously reduced compared with those of patients in the control group, which implied that empagliflozin could effectively reduce blood sugar. The potential pressure-reducing mechanism might be the inhibition of the resorption of sodium ions apart from the reduction of the resorption of glucose, which enabled empagliflozin to reduce plasma volume in the body to lower blood pressure by natriuretic function. Other studies reported that SGLT2 inhibitors effectively reduce patients' blood pressure by reducing the excitability of sympathetic nerve activities, improving endothelial functions and vascular stiffness, and reducing cardiac load. In terms of lipid-lowering functions, TC and TG levels of patients in the two groups were both reduced obviously after the treatment, while LDL-C levels in the two groups showed no obvious changes. Besides, there was no significant difference in lipid-lowering effects between the two groups. Some other studies [31] demonstrated that empagliflozin could result in the increase in LDL-C. The sample size of the research was small with limitations, so the action mechanism of empagliflozin in blood fat needed to be further investigated.

As two new blood sugar-reducing drugs, the effectiveness and safety of the two drugs are evaluated by multiple clinical studies at present. Empagliflozin is the only SGLT2 inhibitor verified to be beneficial to cardiovascular diseases [32]. The results of the large-scale cardiovascular outcome study (CVOT) on empagliflozin [33] showed that the empagliflozin group could reduce the risk of all-cause mortality of arteriosclerotic cardiovascular disease (ASCVD) complicated with diabetes by 32% and that of the incidence of plasma-protamine sulfate-paracoagulation-major adverse cardiovascular events (3P-MACE) by 14%. LEADER series studies also verified that liraglutide was a safe and efficient blood sugar-reducing drug with cardiovascular safety compared with traditional blood sugar-reducing drugs. Besides, it was beneficial to patients in various aspects of clinical treatment.

In the research, diabetes remote medical treatment management system was constructed for both doctors and patients based on the latest IoT technology from the perspective of diabetes management. In addition, the informationization, intelligence and automation of diabetes remote medical treatment management were realized. However, there were still many disadvantages in the research. For instance, only the function of remote medical treatment in T2DM patients was studied in the research with high rate of loss of follow-up visits. Further improvement was needed in the subsequent studies.

## 5. Conclusion

To conclude, the joint treatment by empagliflozin and liraglutide could not only reduce blood sugar effectively with the intervention of IoT-based technology diabetes remote medical treatment management system but also show the advantages in weight loss, blood pressure and blood fat reduction, and low risk in hypoglycemia. Furthermore, it could effectively improve insulin resistance as well as the secretion functions of islet *β* cells of patients and enhance patients' self-management capability. The treatment method provided a new choice for the personalized treatment of overweight/obesity T2DM patients.

## Figures and Tables

**Table 1:** Statistics of general data ($\bar{x}\pm s$).

	*n*	Age (year old)	Gender		Waistline (cm)
			Female	Male	
Control group	25	52.35 ± 12.15	13	12	111.56 ± 9.67
Joint group	25	54.27 ± 11.68	14	11	110.89 ± 8.96
*P* value		0.324	0.241	0.223	0.125

**Table 2:** Comparison of each index in two groups before and after the treatment ($\bar{x}\pm s$).

Groups	*n*	Weight (kg)	BMI (kg/m^2^)	HbA1c (%)	FPG (mmoL/L)	2hPG (mmoL/L)	HOMA-IR
Control group	25						
Before the treatment		74.72 ± 8.54	27.78 ± 1.72	8.55 ± 0.71	10.07 ± 2.25	15.22 ± 3.89	3.76 ± 1.46
After the treatment		71.84 ± 6.78^a^	26.79 ± 1.74^a^	7.70 ± 0.39^a^	8.12 ± 0.73^a^	12.99 ± 2.46^a^	3.12 ± 0.52^a^
Joint group	25						
Before the treatment		74.96 ± 7.43	28.04 ± 2.27	8.40 ± 0.61	10.79 ± 2.99	15.04 ± 3.64	3.67 ± 1.45
After the treatment		68.12 ± 5.46^ab^	25.51 ± 1.82^ab^	6.97 ± 0.51^ab^	7.54 ± 0.78^ab^	10.84 ± 1.58^ab^	2.63 ± 0.67^ab^

Groups	*n*	HOMA-*β*	SBP (mmHg)	DBP (mmHg)	TC (mmoL/L)	TG (mmoL/L)	LDL-cholesterol (C) (mmoL/L)

Control group	25						
Before the treatment		14.55 ± 8.7	140.2 ± 16.61	82.76 ± 7.59	5.92 ± 1.44	2.82 ± 0.96	3.34 ± 0.87
After the treatment		17.96 ± 3.57^a^	138.20 ± 13.84	80.76 ± 5.58	5.16 ± 1.23^a^	2.24 ± 0.63^a^	3.19 ± 0.86
Joint group	25						
Before the treatment		11.99 ± 7.6	136.44 ± 13.70	79.84 ± 7.43	5.50 ± 1.6	3.08 ± 1.27	3.61 ± 0.61
After the treatment		17.30 ± 4.33^a^	129.44 ± 13.22^ab^	75.00 ± 5.41^ab^	4.49 ± 1.12^a^	2.34 ± 0.78^a^	3.66 ± 0.79

## Data Availability

The data used to support the findings of this study are included in the article.
